# Understanding the agreements and controversies surrounding childhood psychopharmacology

**DOI:** 10.1186/1753-2000-2-5

**Published:** 2008-02-08

**Authors:** Erik Parens, Josephine Johnston

**Affiliations:** 1 The Hastings Center, 21 Malcolm Gordon Road, Garrison, New York 10524, USA

## Abstract

The number of children in the US taking prescription drugs for emotional and behavioral disturbances is growing dramatically. This growth in the use of psychotropic drugs in pediatric populations has given rise to multiple controversies, ranging from concerns over off-label use and long-term safety to debates about the societal value and cultural meaning of pharmacological treatment of childhood behavioral and emotional disorders. This commentary summarizes the authors' eight main findings from the first of five workshops that seek to understand and produce descriptions of these controversies. The workshop series is convened by The Hastings Center, a bioethics research institute located in Garrison, New York, U.S.A.

## Introduction

According to Rutter et al., during the industrialized world's postwar period, "as physical health was improving, psychosocial disorders were becoming more frequent [1]." The cause or causes of this increase are debated. In 2000, the US Surgeon General estimated that approximately one in five children and adolescents experience the signs and symptoms of a recognized (DSM-IV) disorder during the course of a year, of whom about 5% experience "extreme functional impairment" [2]. Some more recent studies support this finding, arguing that a majority of disorders begin before 14 years of age [3], with a significant portion already manifest in preschoolers [4].

In parallel developments, the number of children in the US taking prescription drugs for these disorders is growing dramatically [5,6]. Recent trends in psychotropic medication use from large population-based studies show substantial growth in pediatric and adolescent use of antidepressants [7] and stimulants [8]. According to a study by Medco Health Solutions, an organization that monitors drug spending, the numbers of children under 19 years of age who are taking one or more behavioral drugs rose over 20% between 2000 and 2003, with spending on medications to treat attention deficit disorder rising 183%, antidepressants rising 27%, and medications to treat autism and conduct disorders rising more than 60% in that period [9]. Other studies support these findings regarding the upward trend in the use of psychotropic medications in children [10,11]. This trend has given rise to multiple controversies, ranging from concerns over off-label use and long-term safety to debates about the societal value and cultural meaning of pharmacological treatment of childhood behavioral and emotional disturbances [12-16]. While different positions on these controversies are often expressed in the specialist and lay literatures [12,17,18], few attempts have been made to engage with the controversies in order to learn both what they can tell us about the facts and values at issue, as well as whether there are in fact areas of agreement.

## Method

In response to these controversies, The Hastings Center, a bioethics research institute in Garrison, New York applied for and was awarded a Cooperative Agreement Conference grant from the U.S. National Institute of Mental Health. The grant allows the Principal Investigator, Erik Parens, and Co-Principal Investigator, Josephine Johnston to conduct a 3-year project built around five workshops. The 2-day workshops give a highly diverse and distinguished group of approximately 22 practitioners and scholars an opportunity to talk carefully and respectfully over time. Unlike typical conference presentations, workshop presentations are commissioned to build on one another and each session of three presentations is followed by, on average, sixty minutes of sustained debate by participants seated around one table. Two facts about these workshops – that they are interdisciplinary and that they entail face-to-face interaction over time [19] – make them especially well suited to analyzing complex issues and producing new insights.

Studies of interdisciplinary interaction and distributed research groups support this method [20]. Julie Klein and other leading authorities on interdisciplinary projects and processes have written about the epistemic power that a multi-perspective approach brings to the production, critique, and dissemination of knowledge [21-24]. Studies of "distributed" research groups show that the success of collaborative research undertaken by geographically distanced teams depends on regular face-to-face meetings [26] in environments that encourage researchers to treat each other as equals [27]. The NIH Roadmap also supports interdisciplinary research, noting that: "By engaging seemingly unrelated disciplines, traditional gaps in terminology, approach, and methodology might be gradually eliminated. With roadblocks to potential collaboration removed, a true meeting of minds can take place: one that broadens the scope of investigation into biomedical problems, yields fresh and possibly unexpected insights, and may even give birth to new hybrid disciplines that are more analytically sophisticated [25]." The Roadmap's description applies equally well to the combination of scientific and humanistic disciplines in this workshop series.

The first workshop, held in March 2007 in New York City and reported on in this commentary, aimed to produce an overview of most of the major controversies. Each of the next three workshops is focused around a single childhood emotional or behavioral disturbance and considers the major controversies as well as some that are specific to the particular disorder: the second workshop, held in October 2007, was built around a discussion of ADHD; the third and fourth workshops will be built around discussions of pediatric bipolar disorder and depression, respectively. The final workshop will synthesize the first four and will identify emerging issues for further study.

The project's Steering Committee, consisting of the PI and Co-PI together with Benedetto Vitiello (NIMH), Sara Harkness (University of Connecticut), and Steven Hyman (Harvard University), is primarily responsible for leading the project, including selecting workshop participants. Workshop participants are selected based on their accomplishments of direct relevance to the controversies, their willingness to apply themselves afresh to the workshops' on-going conversation, and their willingness to contribute toward one or more products. Some participants will attend all or nearly all of the workshops, while others will attend only one or two workshops depending on their expertise and availability.

The Steering Committee invited participants from many disciplines (including child psychiatry, neurobiology, epidemiology, philosophy, anthropology, and sociology) as well as researchers who emphasize different positions. The first workshop included those who aim to understand childhood emotional and behavioral disturbances in biological terms and those who begin their inquiries at the level of environment and culture. Because researchers emphasize different insights, we invited clinicians who emphasize the effectiveness of psychotropic medications in childhood, those who are concerned about prescription levels, and those who study the effectiveness of non-pharmacological treatments. We also invited researchers who have been part of DSM- and ICD-related efforts to articulate reliable and valid diagnoses and individuals who have written critically about diagnostic categories, as well as researchers who could speak directly to the roles of nature *and *nurture in the emergence of childhood disturbances, the problems of over *and *under-treatment, as well as the problem of stigma *and *the problem of the desire for diagnostic labels.

Ultimately, the project aims to produce a fair description of the interconnected controversies and areas of agreement related to the use of drugs in treating childhood behavioral and emotional disturbances and to identify areas where further conversation and research are required. The project does not seek consensus. Although one or more of these controversies has been addressed by individuals or specialist groups, we have not found publications or other reports by groups including such a wide variety of disciplines and perspectives. We therefore believe that our findings will shed new light on the precise nature of the controversies over the pharmacological treatment of emotional and behavioral disturbances in children, including the areas of agreement and disagreement. For example, it may be that deep disagreement exists over whether it makes a moral difference whether one uses pharmacological or non-pharmacological treatments in children, yet it may be that there is nonetheless widespread agreement that more money and effort should be directed towards establishing stable home and school environments for children. Our findings will then be communicated to various audiences in oral presentation, articles (such as this commentary), a book of essays, a report, and a web-based e-briefing.

## Findings: Agreement and divergence at workshop 1

The commentary you are now reading summarizes the findings from the first of the five workshops. Although all workshop participants (see acknowledgements) have had an opportunity to suggest or request changes to the commentary, it is the sole work of the authors and reflects our understanding of the controversies discussed at the workshop. The commentary identifies 8 major points about which we and the workshop participants (WP) agreed and that we think require recognition within the specialist and lay communities. After identifying a major point of agreement, we then identify areas of disagreement – or, more accurately, areas of differing emphasis. Unless otherwise specified, quotation marks indicate that the words were spoken by participants at the workshop or appeared in their Power Point presentations or handouts.

### 1. Human societies have an obligation to help children (and families) who are suffering from behavioral or emotional disturbances

Different WPs did, however, emphasize different ways in which we should work to relieve suffering from emotional and behavioral disturbances. Some WPs tended to focus on what might be done to help the individual child now. For example, Carol Caruso, who works on behalf of the National Alliance on Mental Illness, focuses on finding ways to help parents to quickly and efficiently ameliorate their child's suffering. She rarely has time to ask about the distal causes of that child's emotional or behavioral disturbance or to speculate about the potential wider cultural or social effects of whatever treatment is chosen.

Other WPs, however, like anthropologist Sara Harkness and developmental psychologist Charles Super, focus on "the relationship between the developing child and the environment [i.e., the culture]" that contributes to producing emotional and behavioral disturbances in children. Harkness and Super referenced studies they have led that examine the cultural belief systems and daily lives of children and parents – with a view to getting a clearer picture of the relationship between different parenting styles and different rates of childhood psychiatric diagnoses.

Though social scientists seek to make descriptive rather than evaluative claims, one reading of the Harkness-Super data is that it supports two related but different evaluative claims about how American culture can produce suffering in children [28]. First, their comparison of American and Dutch parenting styles suggests that Americans are more prone than the Dutch to create environments that over-stimulate children [29] and thus inadvertently create the behaviors that drugs like Ritalin are intended to treat. Second, their comparison of American and Italian parenting styles suggests that Italian parents are less likely to consider the mood of their children to be problematic [30], making them less prone to label their children as developmentally abnormal and thus less likely to inadvertently create the suffering that sometimes attends getting a psychiatric diagnosis or taking medication.

### 2. To understand the emergence of childhood emotional and behavioral disturbances, we need an "ecological" or "systems" or "interactionist" approach – an approach that studies biological and environmental variables as they interact over time

Some WPs emphasized the role of biological variables. When these WPs think of depression, for example, they think first of the important role of *genetic *differences, which has been demonstrated by traditional (twin, adoption, and family) behavioral genetics studies – and they also think of recent findings from molecular genetics and neuroanatomy, which describe correlations between genetic or anatomical and functional differences and differences in mood and behavior [31-33].

Other WPs, however, tended to emphasize the role of environmental variables. For example, pharmacological epidemiologist Julie Zito cited psychiatrist Leon Eisenberg, who said: "All children inherit – along with their parents' genes – their parents, their peers, and the communities they live in" [34]. When these WPs think of depression, they think first of research on the role of *environmental *differences and neuroscience research that shows the role of stress [35]. Psychologist-behavioral geneticist, Julia Kim-Cohen, presented emerging research on gene-environment interactions that aims to help us better understand why, when children are exposed to environmental risks, some go on to develop mental disorders while others do not. This evidence suggests that genes can moderate the impact of environmental "pathogens," such as physical maltreatment, on the risk for developing mental disorders [36-38].

### 3. DSM IV's – and ICD 10's – categorical approach to mental disorders does not represent clinical reality as accurately as would a dimensional approach

At least three WPs (psychiatrists Michael First, Steven Hyman, and Benedetto Vitiello) said that "the reification" of DSM categories is a significant problem. Psychiatrist John Sadler pointed out that the Introduction to DSM IV actually grants that a *dimensional *approach would better reflect clinical reality than the *categorical *one does – that it is usually a serious mistake to speak as if bright lines separate the categories of health and disease or separate categories of disease. Indeed, Michael First, one of DSM IV's editors, said in no uncertain terms that " [there are] no 'zones of rarity' between normal and disorder or between disorders (e.g., schizophrenia – schizoaffective disorder – psychotic mood disorder)." Alas, as Sadler noted, "The introduction to DSM IV is really excellent. The problem is nobody reads it."

Michael First proposed that "Most comorbidity is an artifact of the [DSM] system" and that "as categories are more narrowly defined, more [disorders] are present in the same patient [and thus patients receive more treatments]." Indeed, Benedetto Vitiello showed a slide indicating the extraordinary overlap among the diagnoses ADHD, Tic Disorder, Mood Disorder, Conduct Disorder, and Oppositional Defiant Disorder. He suggested that we do not know whether these comorbidities point toward one underlying psychopathology or many.

Vitiello also pointed out the more general problem that, while DSM diagnoses are reliable across trained raters, the current DSM approach is limited by the fact that it is purely descriptive. The DSM is explicitly a-theoretical and makes no attempt to offer causal explanations of the conditions it describes.

Despite the difficulties and limitations associated with DSM and ICD's categories, some WPs emphasized their usefulness. Philosopher Kenneth Schaffner mentioned that rheumatoid arthritis is a complex and dimensional trait, but that clinicians find the categorization of its types useful. Michael First pointed out that: psychiatrists, like other physicians operating out of the medical model, have to make categorical decisions (whether or not to treat); categories facilitate communication between clinicians; and categories are a central part of the ongoing psychiatric enterprise, including basic research, practice, and drug development. Moreover, First suggested, eliminating the categories would be an administrative nightmare, would require massive retraining of all in the mental health field, and would disrupt research practices. He recommended moving to a hybrid approach, which would integrate dimensions with categories and would, for example, indicate the severity of the disorder and the range of treatments appropriate for different severities.

Others emphasized considerable skepticism about the usefulness of the current categories. Pediatrician William Carey, sociologist Peter Conrad, anthropologist Sara Harkness, developmental psychologist Charles Super, and others worried that the categories are too numerous and wide, and that they unnecessarily and even harmfully bring children with normal temperamental differences within the purview of medicine. Pharmacological epidemiologist Julie Zito suggested that, perhaps due to the DSM's descriptive approach, a biologically-based treatment model had emerged in which the presence of symptoms alone tends to lead to a diagnosis without sufficient attention being paid to the severity of the symptoms and the impairment they cause. She is also concerned that a validity problem continues to plague psychiatry, despite its efforts to embrace a scientific model [39].

### 4. Values play an ineliminable role in the diagnosis of childhood psychiatric disorders

It became clear to us during the workshop that values are ineliminable because, as noted above, human emotions and behaviors are expressed along a continuum – mood, attention, and activity are all dimensional or quantitative traits. As Julie Zito observed, just because we can measure a behavior, label it as disordered, and treat it, does not mean that it "is" a disorder. The precise boundary between normal and abnormal phenotypes must be chosen by us, based on our observation of symptoms and assessments of harmful dysfunction; it is up to us to determine when an individual's suffering rises to the level of warranting treatment.

For example, as child psychiatrist Peter Jensen pointed out, it may be that widely distributed traits such as those associated with ADHD once upon a time conferred an adaptive advantage. Where once those traits may have helped an individual, today they can be sources of suffering or dysfunction. As psychiatrist and neurobiologist Steve Hyman put it, insofar as what counts as a disturbance worth treating always entails judgments about what is harmful for someone, diagnoses are "influenced by professional, social, and cultural values."

Current inter- and intra-national variation in patterns of diagnosis and treatment [40,41] also reflect value differences and not, or not simply, differences in occurrence. To explain varying rates of diagnosis, some WPs emphasized differences across cultures regarding the expectations of developing children. Child psychiatrist Benedetto Vitiello observed that culture may not affect the frequency and presentation of a certain behavior, but it does certainly influence the interpretation of the behavior. And child psychiatrist Jörg Fegert pointed out that the intensity of the desire of parents "to facilitate or even improve the development and the chances of their children" may also vary with culture. Sociologist Ilina Singh added that that even political agendas within psychiatry (e.g. a concern to be not like the USA) might affect diagnostic rates. On the other hand, some WPs emphasized that more children are diagnosed today due to better mental health care. NAMI representative Carol Caruso suggested that more children are diagnosed earlier because we are better at recognizing these disorders earlier.

In response to the discussion of the role of values in making psychiatric diagnoses, some WPs emphasized the role that values play in *all *diagnoses, whether in psychiatry or the rest of medicine. Psychiatrist Michael First observed that there is nothing surprising or unsettling about the fact that psychiatric diagnoses, like other medical diagnoses, entail the value judgment that suffering is bad. Some WPs also emphasized the reasonableness of treating dysfunction wherever we see it, whether we call it a temperamental difference or a disorder (or whether we call the person "bad" or "mad"). Steve Hyman asked, "If someone is suffering, should we care whether its source is a temperamental difference or a disorder? Shouldn't we relieve that suffering if we have the tools?"

Others, however, emphasized that value judgments play a larger role in psychiatry than in other branches of medicine. Former editor of the *New England Journal of Medicine*, Marcia Angell said that "The DSM IV ... is the product of judgments of about 170 experts, but not necessarily supported by published data. Of necessity, these judgments are often subjective. [Psychiatric disorders are] not like cancer or heart failure." This subjectivity – combined with the observation that traits are dimensional – led some WPs to advocate letting natural differences be – being slower to intervene. Pediatrician William Carey, for example, argued that children have a huge variety of temperaments (behavioral styles) and adjustments (behavioral content) and that this variety is normal. But because we lack an adequate available rating system for the dimensions of normal temperament and adjustment, he warned that "given a choice between categorical abnormal diagnosis and nothing, the clinician may be tempted to overuse the abnormal." If a child presents with a normal temperamental or adjustment difference, we should, he suggested, leave the child be – or we should manage the child's behavior with counseling (as opposed to psychotherapy or drugs).

### 5. Rather than be for or against medicalization, we need to get better at distinguishing between good and bad forms of medicalization

As Benedetto Vitiello observed, we can all agree that, to the extent that medicalizing childbirth saves the lives of women and children, it is good; similarly, we can agree that labeling political dissenters as mentally ill (a form of medicalization that occurred in the former Soviet Union) is bad. It was religious studies scholar, Sidney Callahan, who articulated the group's widely shared view that we need to get clearer about the difference between "good" and "bad" forms of medicalization.

Again, though, different WPs emphasized different points. Psychiatrist John Sadler, for example, argued that medicine's primary focus should be to treat *non-moral *problems and that other social institutions (education, religion, criminal justice) should address the *moral *problems that too-often have crept into DSM's and psychiatry's ambit (e.g., Conduct Disorder): As he put it, "The mental health field should draw stricter boundaries between mental disorders and vice." Sadler believes that, as we define more and more moral problems as medical problems, we confuse the public about what he takes to be the fundamental difference between "badness" and "madness," between wrongful or criminal conduct and mental illness. Philosopher Bonnie Steinbock suggested that, whatever the conceptual difficulties with the distinction between "bad" and "mad," it would be pragmatically impossible to give it up entirely, since a criminal justice system requires us to be able to distinguish between criminal behavior – which is generally deserving of punishment – and behavior that, because it is the product of mental disorder, may not be deserving of punishment.

Some WPs, however, emphasized that we should use medicine if it helps achieve our aims, regardless of whether those aims are traditionally within the purview of medicine. Along the lines of psychiatrist Michael First above, psychiatrist Benedetto Vitiello argued: "Our society has decided that pain, suffering, murder, aggression are bad. Getting along with others, respecting the law are good. And these are the same values that medicine has to pursue. In some ways it's irrelevant if disorders are classified as illness or vice."

### 6. Even when child psychiatrists can agree about the boundary between healthy and disordered emotions and behaviors in children, misdiagnosis remains a problem

Even those who whole-heartedly accept the DSM or ICD definitions of a given childhood psychiatric disorder, agree that there are children who need treatment who are not getting it and children who do not need treatment who are. As psychiatrist and neurobiologist Steve Hyman said, "There are some kids who are sick and are ignored and there are some annoying kids who are getting medicalized and we can't tell them apart very well."

Some WPs emphasized that children who do not need stimulants are getting them anyway. Epidemiologist Jane Costello pointed out that, in the Great Smoky Mountains study, "More children without ADHD than with it received prescriptions of stimulants." On the other hand, children who need stimulant treatment are not getting it. Costello also pointed out that another finding of the GSM study is that the percentage of children with ADHD who are *not *receiving medication is too large (28%) [42]. The recent epidemiological study of parent reports by Froehlich et al. suggests that "less than half of children who met DSM-IV ADHD criteria had reportedly had their conditions diagnosed by a health care professional or been treated with medications [43]."

### 7. Once a line is drawn between healthy and disordered emotions and behaviors in children, both pharmacological and non-pharmacological treatments can be appropriate

Some WPs emphasized the similar effects achieved by drugs and psychosocial interventions and argued that it makes no moral difference which kind of intervention we use. As Steve Hyman pointed out, "both psychotropic drugs and lived experience produce long-term changes in the brain that are not well understood." He acknowledges that long-term developmental effects of psychotropic drugs are not known – but pointed out that neither are the long-term effects of no treatment. Nor do we have a good understanding of the effects, or efficacy, of psychosocial interventions. He argued that, in fact, psychosocial interventions can have negative consequences. Hyman suggested that there is a "cultural bias that behavioral interventions are totally benign and less potent" and argued that a preference for non-pharmacological treatment was a symptom of what he called "pharmacological Calvinism," or an unexamined gut feeling about the wrongness of using pharmacological means to treat mental disorders.

Making a related point, psychiatric epidemiologist Jane Costello described a study in which two groups of children were compared over time; one group had a complex psychosocial intervention and the other group had no intervention: the "intervention was multi-systemic therapy, behavioral treatment, psychotherapy, financial support, etc. for five years [44]. In adulthood, on every measure, the intervention group did significantly worse than the non-intervention group."

Others, however, believe that our choice of means to intervene matters morally and that psychosocial and environmental approaches are sometimes preferable because of their net effect on the developing child and/or because they get to the root of the problem rather than merely altering the child to fit a problematic environment. Anthropologist Sara Harkness argued that in order to address suffering we need to consider "treating" the child's environment rather than only treating the child. As developmental psychologist Charlie Super observed: If 80% of children have diarrhea, the answer is not only to give them all medication, but to also treat the problem at the macro-level. In the case of childhood mental disorders, this could include influencing parenting practices and the institutions in which children spend time, like schools. Pediatrician Bill Carey argued that what he calls temperamental differences (what some others may call disorders) "can generally be managed satisfactorily by counseling toward accommodation and improving the interaction and fit, but not by medication or psychotherapy."

In terms of patient and family preferences, child psychiatrist Benedetto Vitiello noted that there is some evidence that, in general, parents would prefer therapy or other non-pharmacological treatment over drugs, although many nonetheless choose drugs because they are considered significantly cheaper and easier to administer. Educational psychologist Roy Martin also noted that in schools "there is enormous pressure for a quick fix; a pill."

### 8. We need to be attentive to the political, economic, legal, institutional realities and health systems in which children's emotional and behavioral disturbances occur and are treated

Some WPs, like pediatrician Kelly Kelleher, emphasized the continued negative effect of the traditional separation of psychiatry from the rest of medicine on health policy and funding for mental health services. There was also concern that, in the face of efforts to integrate mental health care into general medical care and to secure comparable funding for mental health services, the anti-psychiatry movement might be able to prevent the provision of mental health services, including screening of children.

Many WPs also emphasized the need for further research on the efficacy of treatments and the effects and effectiveness of treatments on (developing) children; child psychiatrist Jon McClellan, for instance, argued that finding efficacious treatments remains a large challenge. Epidemiologist Julie Zito argued that further research was required to provide physicians and patients with the information they need to make informed decisions about the risks and benefits of different treatment options. Some also emphasized the need for better training. They saw a lack of expertise in those who are doing much of the diagnosing and treating of children and a lack of necessary tools in pediatric primary care. Steve Hyman mentioned the concern that "many psychotropic drugs are prescribed by primary care physicians who may not have the tools [to do adequate diagnosis]." Educational psychologist Roy Martin expressed a similar concern about the involvement of teachers and schools, observing that "most children are initially referred for interventions for behavioral and learning problems based on teacher perceptions," yet teachers have little training in understanding individual differences. In the absence of expertise, Martin argued, they must of necessity base their decisions on inexplicit norms and, when asked to complete behavior rating scales, risk being influenced by factors not related to the student.

All agreed that the cost of screening and treatment is important and must always be borne in mind. Julie Zito noted that screening for problems with hearing, vision, and diabetes is non-controversial, as is childhood vaccination. Yet in the area of mental health, screening for emotional and behavioral disturbances is far more controversial, partly because of the high false positive rate [45]. There was specific concern that not enough data exist on the costs of treating and not treating, including data that consider the costs to other systems/institutions, such as the cost to the justice system, of *not *treating children with behavioral problems or of choosing one kind of treatment over another. Addressing both cost and quality of care concerns, pediatrician Kelly Kelleher suggested that one reason too few children get the treatment they need is that the current system (paper instruments are used to diagnose and track treatments) is inefficient and should be replaced with computerized risk assessments that display results with clinical guidance. Such an approach would provide faster results, lower variable costs, and more accurate responses from patients [46].

There was also widespread agreement that organizations with economic or ideological commitments can stand in the way of children and families getting the best possible help in dealing with emotional and behavioral disturbances. The Citizens Commission on Human Rights, for example, which is sponsored by the Church of Scientology, opposes many practices in psychiatry including the use of many psychotropic medications. In relation to the pharmaceutical industry, former NEJM editor Marcia Angell observed: "The misuse of psychotropic medications in kids is all too common and I believe if you look behind it you find the pharmaceutical industry to a great extent." According to Angell, with the still relatively new practice of a company giving complex protocols to doctors, which are intended to make one company's product look better than another's, "the research establishment is now essentially bought." Some, however, worry less than others. Psychiatrist Jörg Fegert observed that the studies are so complicated, regulated, and expensive that they cannot be conducted without industry support.

## Conclusion

As the debates about the treatment of childhood emotional and behavioral disturbances grow more common, complex, and public, it is reasonable to expect similar points of agreement and disagreement to emerge. Being on the lookout for them, and remembering that even where there are disagreements there are also points of fundamental agreement, might make those debates more productive in the future than they have been in the past.

As the discussion at the first of our 5 workshop series showed, our understanding of the emergence of complex human traits is in its infancy [47]. Particular and contested values inform decisions about which behaviors and/or emotions deserve treatment and which do not. We should expect the kinds of differing perspectives recorded at this workshop. Some individuals will argue that society can reduce the suffering of children by more aggressively diagnosing and treating them with or without drugs. Others will argue that reducing the suffering of children (and the rest of us) calls for more aggressively expecting and affirming different ways of being a child – that is, eschewing aggressive diagnosing and treating and paying more attention to changing cultural practices and environments. All should agree, however, that what we might call "therapeutic humility" – being clear about the limits of understanding – is called for, as is more research on both the causes of behavioral and emotional disturbances and the most effective and respectful ways of responding to them.

## Authors' contributions

The authors contributed equally to this work and both read and approved the final manuscript.

## References

[B1] Rutter M, Smith D (1995). Psychosocial Disorders in Young People: Time Trends and their Causes.

[B2] US Department of Health and Human Services (1999). Mental Health: A Report of the Surgeon General.

[B3] Kessler RC, Berglund P, Demler O, Robert J, Merikangas KR, Walters EE (2005). Lifetime prevalence and age-of-onset distributions of *DSM-IV *disorders in the National Comorbidity Survey Replication. Arch Gen Psychiatry.

[B4] Egger HL, Angold A (2006). Common emotional and behavioral disorders in preschool children: presentation, nosology, and epidemiology. J Child Psychol Psychiatry.

[B5] Safer DJ, Zito JM, Fine EM (1996). Increased methylphenidate usage for attention deficit disorder in the 1990s. Pediatrics.

[B6] Zito JM, Safer DJ, dosReis S, Gardner JF, Boles M, Lynch F (2000). Trends in the prescribing of psychotropic medications to preschoolers. JAMA.

[B7] Delate T, Gelenberg AJ, Simmons VA, Motheral BR (2004). Trends in the use of antidepressants in a national sample of commercially insured pediatric patients, 1998 to 2002. Psychiatric Services.

[B8] Habel LA, Schaefer CA, Levine P, Bhat AK, Elliott G (2005). Treatment with stimulants among youths in a large California health plan. J Child Adolesc Psychopharmacol.

[B9] Medco Health Solutions. http://phx.corporate-ir.net/phoenix.zhtml?c=131268&p=irol-newsArticle&ID=571791&highlight=.

[B10] Olfson M, Marcus SC, Weissman MM, Jensen PS (2002). National trends in the use of psychotropic medications by children. J Am Acad Child Adolesc Psychiatry.

[B11] Zito JM, Safer DJ, dosReis S, Gardner JF, Magder L, Soeken K, Boles M, Lynch F, Riddle MA (2003). Psychotropic practice patterns for youth: a 10-year perspective. Arch Pediatr Adolesc Med.

[B12] Timimi S, Taylor E (2004). ADHD is best understood as a cultural context: for and against. British Journal of Psychiatry.

[B13] Coghill D (2004). Use of Stimulants for Attention Deficit Hyperactivity Disorder. BMJ.

[B14] Biederman J (2005). Attention-deficit/hyperactivity disorder: a selective overview. Biological Psychiatry.

[B15] Singh I (2004). Doing their jobs: mothering with Ritalin in a culture of mother-blame. Social Science and Medicine.

[B16] Conrad P, Potter D (2000). From Hyperactive Children to ADHD Adults: Observations on the Expansion of Medical Categories. Social Problems.

[B17] Olfman S, (Ed) (2006). No Child Left Different.

[B18] Jensen P, Knapp P, Mrazek D (2006). Toward a New Diagnostic System for Child Psychopathology: Moving Beyond DSM.

[B19] Nardi BA, Whittaker S, Hinds P, Kiesler S (2002). The Place of Face-to-Face Communication in Distributed Work. Distributed Work.

[B20] Boix-Mansilla V, Gardner H (2004). Assessing interdisciplinary work at the frontier: An empirical exploration of "symptoms of quality.

[B21] Klein JT (1990). Interdisciplinarity: History, Theory, and Practice.

[B22] Moran J (2002). Interdisciplinarity.

[B23] Nowotny H, Scott P, Gibbons M (2001). Re-thinking Science Knowledge and the Public in an Age of Uncertainty.

[B24] Turner B (1990). The interdisciplinary curriculum: from social medicine to postmodernism. Sociology of Health and Illness.

[B25] National Institutes of Health. Interdisciplinary Research. http://nihroadmap.nih.gov/interdisciplinary/index.asp.

[B26] Epstein SL, Derry SJ, Gernsbacker MA (2005). Making interdisciplinary collaboration work. Interdisciplinary collaboration: an emerging cognitive science.

[B27] Schunn C, Crowley K, Okada T, Hinds P, Kiesler S (2002). What Makes Collaborations Across a Distance Succeed? The Case of the Cognitive Science Community. Distributed Work.

[B28] Harkness S, Moscardino U, Rios Bermudez M, Zylicz PO, Welles-Nystrom B, Blom M, Parmar P, Axia G, Super CM (2006). Mixed methods in international collaborative research: The experiences of the International Study of Parents, Children, and Schools. Cross-Cultural Research.

[B29] Harkness S, Super CM, Moscardino U, Rha JH, Blom M, Huitrón B, Johnston C, Sutherland M, Hyun OK, Axia G, Palacios J (2007). Cultural models and developmental agendas: Implications for arousal and self-regulation in early infancy. Journal of Developmental Processes.

[B30] Super C, Harkness S, Axia G, Welles-Nyström B, Palacios J, Zylicz PO, McGurk H Culture, temperament, and the 'difficult child.'. European Journal of Developmental Science.

[B31] Sapolsky RM (2000). Glucocorticoids and Hippocamapl Atrohpy in Neuropsychiatric disorders. Arch Gen Psychiatry.

[B32] Duman RS (2002). Structural alterations in depression: cellular mechanisms underlying pathology and treatment of mood disorders. CNS Spectrums.

[B33] Sullivan PF, Neale MC, Kendler KS (2000). Genetic epidemiology of major depression: review and meta-analysis. American Journal of Psychiatry.

[B34] Eisenberg L (1999). Experience, Brain, and Behavior: The Importance of a Head Start. Pediatrics.

[B35] Burke HM, Davis MC, Otte C, Mohr DC (2005). Depression and cortisol responses to stress: a meta-analysis. Psychoneuroendocrinology.

[B36] Kim-Cohen J, Caspi A, Taylor A, Williams B, Newcombe R, Craig I, Moffitt TE (2006). MAOA, maltreatment, and gene-environment interaction predicting children's mental health: New evidence and a meta-analysis. Molecular Psychiatry.

[B37] Caspi A, Sugden K, Moffitt TE, Taylor A, Craig IW, Harrington H (2003). Influence of life stress on depression: Moderation by a polymorphism in the 5-HTT gene. Science.

[B38] Moffitt TE, Caspi A, Rutter M (2005). Strategy for investigating interactions between measured genes and measured environments. Arch Gen Psychiatry.

[B39] Eriksen K, Kress VE (2005). Beyond the DSM Story.

[B40] Polanczyk G, de Lima MS, Horta BL, Biederman J, Rohde LA (2007). The worldwide prevalence of ADHD: a systematic review and metaregression analysis. American Journal of Psychiatry.

[B41] LeFever GB, Dawson KV, Morrow AL (1999). The extent of drug therapy for attention-deficit/hyperactivity disorder among children in public schools. American Journal of Public Health.

[B42] Angold A, Erkanli A, Egger HL, Costello EJ (2000). Stimulant treatment for children: a community perspective. J Am Acad Child Adolesc Psychiatry.

[B43] Froehlich ET, Lanphear BP, Epstein JN, Barbaresis WJ, Katusic SK, Kahn RS (2007). Prevalence, recognition, and treatment of Attention-Deficit/Hyperactivity Disorder in a national sample of US children. Arch Pediatr Adolesc Med.

[B44] McCord J (1978). A thirty-year follow-up of treatment effects. Am Psychol.

[B45] Shaffer D, Scott M, Wilcox H, Maslow C, Hicks R, Lucas CP, Garfinkel R, Greenwald S (2004). The Columbia Suicide Screen: validity and reliability of a screen for youth suicide and depression. J Am Acad Child Adolesc Psych.

[B46] National Initiative for Improving Children's Healthcare Quality, Improving Care for Children with ADHD. http://www.nichq.org/NR/rdonlyres/67EFB37E-37DC-4868-A4DB-28BC2C82B532/3816/ADHDDissemBook.pdf.

[B47] Hyman SM (2007). Can neuroscience be integrated into the DSM?. Nature Reviews Neuroscience.

